# Refining Robotic Extravesical Ureteral Reimplantation: Impact of Ureteral Adventitia Inclusion and Distal-First Detrusorraphy

**DOI:** 10.3390/jcm15031221

**Published:** 2026-02-04

**Authors:** Sangmin Lee, Jaeyoung Cho, Kyenghyun Nam, Kun Suk Kim, Sang Hoon Song

**Affiliations:** 1 Department of Urology, Asan Medical Center, University of Ulsan College of Medicine, Seoul 05505, Republic of Korea; sangminlee92@naver.com (S.L.); tactin24@amc.seoul.kr (J.C.); d251469@amc.seoul.kr (K.N.); kunsukkim2@gmail.com (K.S.K.); 2 Department of Urology, Uijeongbu Eulji Medical Center, Eulji University, Uijeongbu-si 11759, Republic of Korea

**Keywords:** vesicoureteral reflux, robotic surgical procedures, reimplantation

## Abstract

**Background**: Robot-assisted laparoscopic ureteral reimplantation via an extravesical approach (RALUR-EV) is an established minimally invasive option for vesicoureteral reflux (VUR); however, surgical success remains variable, and detrusorraphy technique is a key determinant of reflux resolution. This study evaluated whether a refined detrusorraphy technique is associated with improved surgical outcomes following RALUR-EV. **Methods**: We retrospectively reviewed patients who underwent RALUR-EV performed by a single surgeon between August 2013 and February 2023. A technique modification introduced in November 2021 incorporated ureteral adventitia inclusion during detrusorraphy and a distal-first detrusorraphy suture. Patients were divided into two groups according to the surgical period. Surgical success was defined as radiographic resolution of VUR on postoperative voiding cystourethrography without ureteral obstruction. **Results**: A total of 62 patients (96 ureters) were included. The modified technique group demonstrated significantly higher surgical success rates than the conventional group at both the ureter level (97.8% vs. 76.5%, *p* = 0.002) and the patient level (96.6% vs. 69.7%, *p* = 0.006). On patient-level multivariable analysis, the modified detrusorraphy technique was independently associated with a reduced risk of surgical failure. **Conclusions**: A refined detrusorraphy technique is associated with improved early radiographic success after RALUR-EV without increasing perioperative morbidity.

## 1. Introduction

Vesicoureteral reflux (VUR) poses significant risks to renal health, potentially leading to renal dysfunction and scarring as a result of repeated febrile urinary tract infections (UTIs) and pyelonephritis [1,2]. As of now, open ureteral reimplantation (OUR) and endoscopic surgical therapy remain the most widely used surgical options for the management of VUR [3,4]. OUR has historically served as a reference standard for durable radiographic resolution, while endoscopic therapy provides a less invasive approach but with variable success, particularly in high-grade reflux [3,5].

With the increasing application of robotic surgery in pediatric urology, robot-assisted laparoscopic ureteral reimplantation via an extravesical approach (RALUR-EV) has emerged as an effective minimally invasive alternative for OUR [6]. RALUR-EV has been shown to achieve success rates comparable to those of OUR with favorable perioperative outcomes in multiple studies [7,8,9]. RALUR-EV was also associated with decreased morbidity, such as bladder spasms, hematuria, and pain, along with shorter hospital stays when compared to OUR [10,11,12].

Over the past two decades, numerous outcome studies of RALUR-EV have been published, showing a gradual increase in success over time [13]. Nevertheless, variability in outcomes persists across centers, and technical factors related to detrusor tunnel creation and detrusorraphy continue to represent key determinants of reflux resolution and the risk of ureteral obstruction [14].

Several technical refinements have been proposed to address these challenges. Casale et al. introduced a nerve-sparing technique that identifies the pelvic plexus to prevent damage to that area, intending to avoid postoperative bladder dysfunction [15]. Several publications have introduced ureteral advancement and ‘top-down’ suturing techniques as innovative approaches to enhance surgical outcomes [16,17]. Additionally, Gundeti et al. released a paper presenting a standardized technique modification that demonstrated improvements in outcomes [18]. These technical modifications, particularly in the detrusorraphy process, hold significant importance in enhancing the success rate of RALUR-EV.

Building on prior studies of technical modifications, our research team introduced a refined detrusorraphy technique for RALUR-EV in 2021, which involved the inclusion of ureteral adventitia in detrusorraphy and a distal-first detrusorraphy suture. This study aimed to evaluate the association between these technical modifications and the success rate of RALUR-EV.

## 2. Materials and Methods

Following institutional review board approval (IRB No. 2025-0100), we retrospectively reviewed patients who underwent RALUR-EV performed by a single surgeon (SH Song) at Asan Medical Center in Seoul, Korea, from August 2013 through February 2023. Indications for RALUR-EV included persistent or exacerbating primary VUR, breakthrough UTIs, and/or progressive renal scarring despite ongoing antibiotic prophylaxis. Our key technique modification was introduced in November 2021, prompting the division of patients into two groups based on this timeline for comparative analysis of operative and postoperative outcomes. Patients with concurrent bladder bowel dysfunction (BBD) were actively managed with behavioral and pharmacological treatments both before and after surgical treatment.

### 2.1. Diagnostic Evaluation

For VUR assessment, our institution used a voiding cystourethrogram (VCUG), kidney and bladder ultrasonography (KUS), and dimercaptosuccinic acid (DMSA) scan. A follow-up VCUG was conducted one to three months after surgery, and a KUS was performed one month after surgery, in accordance with the institutional postoperative imaging protocol. The analysis included only patients who underwent RALUR-EV for VUR and had both preoperative and postoperative VCUG assessments. Surgical success was defined as the radiographic resolution of VUR without evidence of ureteral obstruction.

### 2.2. Surgical Technique

All surgical procedures were performed by using da Vinci Si, Xi, or X Surgical Systems (Intuitive Surgical, Sunnyvale, CA, USA). Patient positioning was supine, optionally with lithotomy, and a 30-degree Trendelenburg tilt was applied. The camera trocar (8 or 12 mm) was placed through the upper edge of the umbilicus. After achieving pneumoperitoneum, two robotic trocars (5 or 8 mm) were inserted under direct vision. The skin incisions were made at the Pfannenstiel incision level on both sides. The trocars were angled cranially during insertion to facilitate a higher fascial incision, enhancing both cosmetic outcomes and the robotic operational space.

The ureter was identified at the pelvic brim’s edge, leading to a peritoneal incision above the ureter. Subsequently, the ureter was mobilized from the level of the vas deferens or uterine artery down to the ureteral hiatus without causing trauma. At the level of the ureteral hiatus, the dissection was conducted clockwise from the 9 o’clock to the 3 o’clock area, preserving the lower half of the hiatal circle. The dissection was carefully conducted close to the ureter to preserve the adjacent neurovascular bundle positioned posterolateral and avert potential voiding issues, particularly in bilateral cases.

A percutaneous hitch stitch was applied at the bladder dome to facilitate the creation of the detrusor tunnel by ensuring adequate space in the posterior bladder area. Detrusorotomy was performed by dividing the detrusor muscle straightly in a superior-medial direction until the bladder mucosa was identified, ensuring a 5:1 ratio of tunnel length to ureter diameter. Detrusor flaps were delicately separated from the mucosa with meticulous dissection, minimizing cauterization near the ureteral hiatus.

### 2.3. Description of the Technical Modification

Patients were divided into two groups based on the timing of surgery relative to the introduction of the technical modification in November 2021. In both groups, an apical ureter hitch suture (5–0 absorbable Vicryl) was initially placed at the superior aspect of the detrusorotomy to stabilize the ureter and minimize ureteral manipulation during subsequent detrusorraphy. In Group 1, detrusorraphy was performed using interrupted sutures in a conventional top-down fashion without intentional inclusion of the ureteral adventitia.

**Figure 1:**
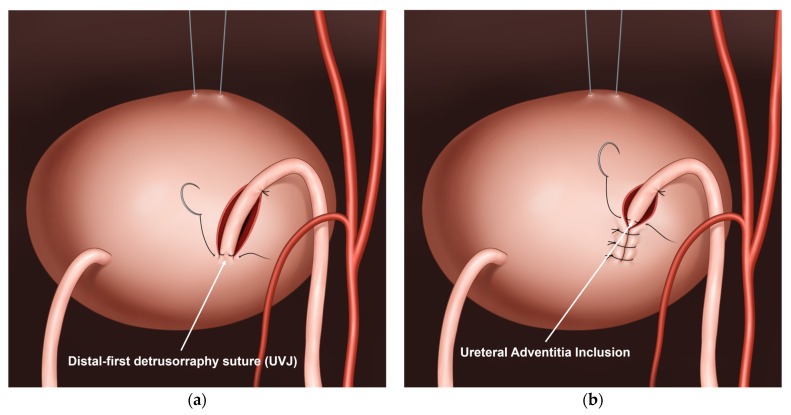
Key elements of the modified detrusorraphy technique. (**a**) Distal-first detrusorraphy suture placed at the ureterovesical junction; (**b**) Ureteral adventitia inclusion during interrupted detrusorraphy, with avoidance of penetration into the ureteral muscular layer.

In Group 2, detrusorraphy was modified to include two key elements: (1) placement of a distal-first detrusorraphy suture using 3–0 or 4–0 absorbable Vicryl at the ureterovesical junction, and (2) intentional inclusion of the ureteral adventitia in each interrupted detrusorraphy suture performed in a bottom-up fashion (Figure 1). The initial detrusorraphy suture was deliberately placed at the distal end of the resulting detrusor tunnel near the ureterovesical junction, securing distal tunnel closure at a stage when visualization and working space were optimal.

For adventitial inclusion, sutures were placed to incorporate only a thin layer of the ureteral adventitia while deliberately avoiding penetration of the ureteral muscular layer. This approach was intended to promote stable ureter–tunnel alignment while minimizing the risk of excessive compression, kinking, or ischemia. Ureteral advancement sutures were not used in either group.

After completion of detrusorraphy, the bladder was partially filled with saline to confirm the absence of mucosal injury, ureteral angulation, or kinking. The peritoneum was then closed, and a urethral catheter was routinely maintained for one to two days postoperatively.

### 2.4. Statistical Analysis

Continuous variables were presented as median (range) and analyzed using the Mann–Whitney U test. Categorical variables were summarized by count and percentage and evaluated through Fisher’s exact test or Pearson’s chi-squared test. Radiographic failure rates at both the patient and ureter levels were summarized descriptively by group.

To identify factors associated with surgical failure, patient-level multivariable logistic regression analysis was performed, with surgical failure defined as persistence of VUR on postoperative VCUG. Odds ratios (OR) with 95% confidence intervals (CI) were reported. Patient-level analysis was designated as the primary inferential approach to avoid non-independence arising from bilateral cases.

Supplementary ureter-level analyses were conducted using generalized estimating equations with patient-level clustering as sensitivity analyses. In addition, a Firth penalized logistic regression was performed at the patient level as a sensitivity analysis to address sparse failure events.

All statistical analyses were performed using R Statistical Software (version 4.3.1, R Core Team, Vienna, Austria), and *p*-values < 0.05 were considered statistically significant.

## 3. Results

During the study period, 67 patients underwent RALUR-EV. Of these, five patients were excluded due to the absence of postoperative VCUG assessments, resulting in a final study cohort of 62 patients (33 in Group 1 and 29 in Group 2). Among the 62 patients, 34 (54.8%) underwent bilateral surgery, resulting in the inclusion of 96 ureters in the analysis (51 ureters in Group 1 and 45 ureters in Group 2) (Table 1).

The median age of patients at the time of surgery was 4.3 years, ranging from 0.3 to 46.7 years. There was a significant difference in median age between the groups. Group 1 patients were significantly older than Group 2 patients (median 5.9 vs. 1.6 years, *p* = 0.005). Gender, body mass index (BMI), prevalence of previous injection therapy, BBD, laterality of VUR, and differential renal function were comparable between the groups. The mean grade of preoperative VUR across the cohort was 3.66, with no significant difference in the distribution of preoperative VUR grades between the groups (*p* = 0.625). Ureters exhibiting VUR grades 1–2 were reimplanted only in individuals with high-grade reflux on the contralateral side.

Surgical success rates at both the ureter and patient levels differed between groups (Table 2). The total operative time ranged from 72 to 320 min, with a median duration of 151.5 min. There was no statistically significant difference in operative time for unilateral RALUR-EV between the groups. However, for bilateral RALUR-EV, Group 2 had a significantly shorter median operative time compared to Group 1 (147.5 min vs. 176 min, *p* < 0.001). All procedures were conducted without intraoperative complications, and minimal blood loss was reported.

Radiographic failure rates at both the patient and ureter levels are summarized in Table 3. Reoperations were required in 7 patients (11.3% of the total cohort), with 6 patients (18.2%) in Group 1 and 1 patient (3.5%) in Group 2 requiring reoperations (*p* = 0.109). The median length of hospital stay was consistent across groups at 2.0 days with no significant difference (*p* = 0.921). The median follow-up length was significantly longer in Group 1 at 26 months (range: 3–99 months) compared to 17 months in Group 2 (range: 1–24 months) (*p* = 0.018).

Postoperative complications within 30 days, classified by Clavien–Dindo grade, were comparable between groups (*p* = 0.149). Postoperative complications included febrile UTIs (n = 5, 8.1%), urinary retention (n = 2, 3.2%), and flank pain uncontrolled with analgesics (n = 2, 3.2%). In Group 1, four patients developed febrile UTIs postoperatively compared to one patient in Group 2; four of these patients required readmission. Of the two patients who experienced urinary retention, one had an underactive bladder prior to surgery, and the other underwent bilateral RALUR-EV. Both patients required Foley catheter reinsertion but subsequently achieved successful self-voiding after catheter removal during outpatient follow-up. In Group 2, two patients underwent ureteral stent insertion due to severe flank pain. One patient was found to have a ureteric stone and subsequently received extracorporeal shockwave lithotripsy, while the other was identified with recurrent partial obstruction at the ureterovesical junction.

Patient-level multivariable logistic regression analysis identified factors associated with surgical failure (Table 4). The modified surgical technique (Group 2) was independently associated with a significantly lower risk (OR, 0.09; 95% CI, 0.01–0.86; *p* = 0.037). Age, gender, bilaterality of surgery, prior injection therapy, BBD, total operative time, and preoperative VUR grade were not significantly associated with surgical failure. As a sensitivity analysis, a ureter-level clustered analysis using generalized estimating equations was performed to account for within-patient correlation, and Group 2 was associated with a significantly lower risk of surgical failure in both unadjusted and adjusted models (Supplementary Table S1). We additionally performed a Firth penalized logistic regression as a sensitivity analysis due to sparse failures; the direction of the group effect was consistent (Group 2 vs. Group 1: OR 0.14, 95% CI 0.01–0.75, *p* = 0.02).

## 4. Discussion

The present study evaluated the impact of our technical modification in RALUR-EV on surgical outcomes for VUR. We observed higher radiographic success rates in patients treated with the modified technique compared with the conventional approach, increasing from 76.5% in the earlier cohort to 97.8% after implementation of the modification. This enhancement is particularly relevant given that the majority of patients had VUR of Grade 3 or higher (91.7%). The success rates achieved with the modified technique are comparable to those reported for OUR, which remains the reference standard for VUR treatment.

With the emergence of robotic surgery and advancements in pediatric urology, the robotic approach has evolved into a more feasible minimally invasive therapeutic option for VUR. Compared to OUR, robotic surgery offers advantages such as reduced invasiveness, improved cosmetic outcomes, and a shorter learning curve compared to the traditional laparoscopic approach, facilitating its adoption in clinical practice [9,19]. In the early stages of robotic surgery, there were papers on RALUR procedures via the intravesical approach [11,20]. However, this approach is more technically demanding than the extravesical approach, encountering challenges in maintaining pneumovesicum and performing the procedure in young children with small bladder sizes [21]. Consequently, the extravesical approach (RALUR-EV) has become the preferred method among pediatric urologists performing robotic surgery.

Over time, a variety of technical refinements have been proposed to optimize outcomes of RALUR-EV. For instance, the ‘top-down’ suturing technique introduced by Silay et al. minimizes ureter manipulation and reduces the risk of injury by superiorly placing the first detrusor tunnel stitch [17]. Nevertheless, technical challenges remained, particularly during placement of the distal stitch near the ureteral hiatus, where visualization and working space are inherently limited. In this context, incorporation of an apical ureter hitch suture into the detrusorraphy technique offered an approach to improve visualization of the detrusor muscle trough during subsequent suturing. This allowed more consistent implementation of a distal-first detrusorraphy suture within a bottom-up sequence, addressing a recognized technical limitation of conventional top-down approaches.

Our technique should be interpreted as a refinement of previously described approaches rather than a fundamentally new procedure, particularly in relation to the LUAA technique reported by Gundeti et al. [18]. This method is characterized by a 4–5 cm submucosal detrusor tunnel (L), a U stitch (U), placement of a permanent apical stay stitch (A), and inclusion of ureteral adventitia (A) during detrusorraphy.

Although our approach shares conceptual similarities with the LUAA technique, several key technical differences exist. Unlike LUAA, our technique does not involve Y-shaped dissection at the ureterovesical junction and does not require placement of a separate U stitch. Instead, detrusorraphy is initiated with a distal-first detrusorraphy suture placed directly at the ureterovesical junction, securing distal tunnel closure at a stage when visualization and working space are optimal and thereby avoiding the visualization constraints of a conventional top-down sequence [16].

In addition, whereas the LUAA technique employs a fixed 4–5 cm submucosal detrusor tunnel, our approach adheres to the traditional 5:1 tunnel length–to–ureter diameter ratio, allowing tunnel length to be individualized according to ureteral size. We also used an absorbable apical stay stitch (5–0 Vicryl) rather than a permanent suture. Despite these technical differences, intentional inclusion of the ureteral adventitia was preserved, reflecting a shared principle aimed at optimizing ureter–tunnel integration and distal tunnel competence.

We acknowledge that the relatively lower outcomes observed in Group 1 may raise concerns regarding learning curve effects, particularly given the shorter operative time in Group 2. However, learning curve effects alone do not fully explain the observed outcome disparity. Chronological analysis within Group 1 showed no improvement in success over time; instead, ureter-based success declined from approximately 89% to 64%, coinciding with increasing case complexity. This pattern suggests inherent limitations of the conventional top-down detrusorraphy approach and supports the interpretation that the improved outcomes in Group 2 reflect not only accumulated experience but also the effect of technique modification.

The improved surgical outcomes observed with the modified technique appear to be associated with the complementary application of distal-first detrusorraphy and controlled adventitial inclusion. Initiating detrusorraphy with a distal-first suture allows secure closure of the ureterovesical junction at a stage of optimal visualization, addressing a known vulnerability of conventional top-down approaches in which distal closure is deferred. Controlled inclusion of a thin layer of the ureteral adventitia further promotes stable ureter–tunnel alignment while avoiding muscular penetration, thereby minimizing the risks of kinking, ischemia, or functional obstruction.

This technical modification not only enhanced surgical success rates but also maintained patient safety, with no intraoperative complications, minimal blood loss, and similar hospital stays across groups. The low and comparable incidence of postoperative complications, assessed via the 30-day Clavien–Dindo scale, underscores the modification’s safety. Although one patient in Group 2 developed partial obstruction at the ureterovesical junction, this finding highlights the importance of meticulous suture placement and avoidance of excessive compression during detrusorraphy.

An additional consideration is the age difference between the two groups, with patients in Group 1 being older than those in Group 2. Although age-related factors have been reported to influence outcomes in certain minimally invasive techniques [5], age was not a significant predictor of surgical failure in multivariable analysis, whereas the surgical group remained independently associated with outcome, indicating that the observed differences cannot be explained by age alone.

With respect to postoperative management of surgical failures in Group 1, failure was defined radiographically based on postoperative VCUG. Among the 10 patients classified as failures, four had low-grade (grade I–II) persistent reflux and were managed conservatively without additional intervention, while the remaining six patients underwent secondary endoscopic injection therapy. All patients remained clinically stable during follow-up without further need for surgical intervention.

Despite these promising results, several limitations warrant consideration. First, the retrospective nature of the study and the relatively small sample size may limit the generalizability of the findings. Second, the median follow-up period of 19 months and the use of early postoperative VCUG as the primary endpoint may not fully capture late recurrent reflux or delayed ureteral obstruction. Third, this study represents a historical comparison cohort performed by a single surgeon, and outcomes may have been partially influenced by temporal effects and increasing surgical experience, although learning-curve effects alone were unlikely to account for the observed differences. In addition, five patients who underwent RALUR-EV during the study period were excluded from the final analysis due to the absence of postoperative VCUG, which may introduce a degree of selection bias. Finally, because distal-first detrusorraphy and controlled adventitial inclusion were introduced simultaneously, the individual contribution of each component could not be isolated within the current study design.

## 5. Conclusions

This study demonstrates that a refined detrusorraphy technique incorporating intentional inclusion of the ureteral adventitia combined with a distal-first detrusorraphy suture is associated with significantly improved early radiographic success following RALUR-EV, without increasing perioperative morbidity. Although this approach represents a refinement rather than a fundamentally new procedure, the findings suggest that targeted modification of detrusorraphy technique can meaningfully influence surgical outcomes in RALUR-EV. Further multicenter studies with longer follow-up are warranted to validate the durability and generalizability of this technical refinement and to establish standardized surgical strategies for optimizing RALUR-EV outcomes.

## Figures and Tables

**Table 1:** Baseline Characteristics of Patients and Ureters Undergoing Robot-Assisted Laparoscopic Ureteral Reimplantation via an Extravesical Approach.

Characteristic	Overall (n = 62)	Group 1 (n = 33)	Group 2 (n = 29)	*p*-Value
Age at surgery, years (range)	4.3 (0.3–46.7)	5.9 (0.3–46.7)	1.6 (0.4–34.7)	0.005
Gender, n (%)				0.599
M	32 (51.6%)	16 (48.5%)	16 (55.2%)	
F	30 (48.4%)	17 (51.5%)	13 (44.8%)	
Weight, kg (range)	15.7 (6.5–89.3)	20.0 (6.6–89.3)	11.3 (6.5–59.5)	0.004
Body mass index, kg/m2 (range)	17.2 (13.5–28.3)	17.1 (13.5–28.3)	17.2 (14.0–26.0)	0.843
Previous injection therapy, n (%)	6 (9.7%)	5 (15.2%)	1 (3.4%)	0.201
Bladder-bowel disorder, n (%)	21 (33.9%)	13 (36.4%)	9 (31.0%)	0.658
Laterality, n (%)				0.946
Right	12 (19.4%)	6 (18.2%)	6 (20.7%)	
Left	16 (25.8%)	9 (27.3%)	7 (24.1%)	
Bilateral	34 (54.8%)	18 (54.5%)	16 (55.2%)	
Total ureters, n (%)	96 (100%)	51 (53.1%)	45 (46.9%)	
Differential renal function, % (range)	46.0 (6.0–100.0)	46.0 (6.0–94.0)	45.8 (11.0–100.0)	0.581
Preoperative VUR grade, n (%)				0.625
1	1 (1.0%)	1 (2.0%)	0 (0.0%)	
2	7 (7.3%)	4 (7.8%)	3 (6.7%)	
3	30 (31.3%)	17 (33.3%)	13 (28.9%)	
4	44 (45.8%)	20 (39.2%)	24 (53.3%)	
5	14 (14.6%)	9 (17.7%)	5 (11.1%)	
Mean grade	3.66	3.65	3.69	

**Table 2:** Postoperative outcomes by Group.

	Overall	Group 1	Group 2	*p*-Value
Total operative time, min (range)	151.5 (72–320)	166 (79–320)	145 (72–305)	<0.001
Unilateral	129.5 (72–305)	131 (79–235)	128 (72–305)	0.612
Bilateral	159.5 (98–320)	176 (115–320)	147.5 (98–180)	<0.001
Length of Stay, days (range)	2.0 (1.0–7.0)	2.0 (1.0–7.0)	2.0 (2.0–5.0)	0.921
Complications (30 days), n (%)				0.149
Clavien grade 0	45 (72.6%)	22 (66.7%)	23 (79.3%)	
Clavien grade 1	9 (14.5%)	6 (18.2%)	3 (10.3%)	
Clavien grade 2	6 (9.7%)	5 (15.1%)	1 (3.4%)	
Clavien grade 3	2 (3.2%)	0 (0.0%)	2 (6.9%)	
Postoperative febrile UTI, n (%)	5 (8.1%)	4 (12.1%)	1 (3.4%)	0.360
Urinary retention, n (%)	2 (3.2%)	1 (3.0%)	1 (3.4%)	>0.999
Readmission within 30 days, n (%)	5 (8.1%)	3 (9.1%)	2 (6.9%)	>0.999
Surgical success (patients), n (%)	51 (82.3%)	23 (69.7%)	28 (96.6%)	0.006
Surgical success (ureters), n (%)	83 (86.5%)	39 (76.5%)	44 (97.8%)	0.002
Reoperations, n (%)	7 (11.3%)	6 (18.2%)	1 (3.5%)	0.109
Follow-up length, months (range)	19 (1–99)	26 (3–99)	17 (1–24)	0.018

**Table 3:** Radiographic failure rates by group at the patient and ureter levels.

Level	Group 1	Group 2
Patient-level failure	10/33 (30.3%)	1/29 (3.4%)
Ureter-level failure	12/51 (23.5%)	1/45 (2.2%)

**Table 4:** Factors Associated with Surgical Failure at the Patient Level: Multivariable Logistic Regression Analysis.

Factor	Odds Ratio	95% Confidence Interval	*p*-Value
Age	1.02	0.95, 1.09	0.660
Gender (Female)	0.24	0.03, 1.81	0.165
Group 2	0.09	0.01, 0.86	0.037
Bilateral RALUR-EV	0.54	0.09, 3.16	0.498
Previous injection therapy	3.56	0.31, 40.63	0.306
Bladder-bowel dysfunction	2.07	0.34, 12.49	0.089
Total operative time	1.00	0.98, 1.02	0.812
Preoperative VUR grade (Worst)	1.51	0.35, 6.48	0.576

## Data Availability

Data is unavailable due to privacy or ethical restrictions.

## Supplementary Materials

The following supporting information can be downloaded at: https://www.mdpi.com/article/10.3390/jcm15031221/s1, Table S1: Ureter-Level Clustered Analysis of Surgical Failure Using Generalized Estimating Equations (Patient-level clustering).

## Abbreviations

The following abbreviations are used in this manuscript:

RALUR-EV	robot-assisted laparoscopic ureteral reimplantation via an extravesical approach
VUR	Vesicoureteral reflux
UTIs	urinary tract infections
OUR	open ureteral reimplantation
BBD	bladder bowel dysfunction
VCUG	voiding cystourethrogram
KUS	kidney and bladder ultrasonography
DMSA	dimercaptosuccinic acid
OR	Odds ratio
CI	Confidence interval
BMI	body mass index
