# Supplementary material for: Refining Robotic Extravesical Ureteral Reimplantation: Impact of Ureteral Adventitia Inclusion and Distal-First Detrusorraphy
Source: J Clin Med. 2026 Feb 4;15(3):1221. doi: 10.3390/jcm15031221 (PMC12898208; doi:10.3390/jcm15031221)
Supplement: Supplementary file 1 [file jcm-15-01221-s001.zip › jcm-4095205-supplementary.pdf]

Supplementary Table S1. Ureter-Level Clustered Analysis of Surgical Failure Using Generalized Estimating Equations (Patient-level clustering)

**Model**	**Factor**	**Odds Ratio**	**95% Confidence Interval**	**p-value**
Unadjusted	Group 2	0.08	0.01, 0.70	0.022
Adjusted	Group 2	0.08	0.01, 0.95	0.044
Adjusted	Age (years)	1.02	0.98, 1.06	0.407
Adjusted	Gender (female)	0.37	0.08, 1.78	0.207
Adjusted	Preoperative VUR grade	1.46	0.74, 2.89	0.292
